# High-frequency ultrasound for intraoperative margin assessments in breast conservation surgery: a feasibility study

**DOI:** 10.1186/1471-2407-11-444

**Published:** 2011-10-12

**Authors:** Timothy E Doyle, Rachel E Factor, Christina L Ellefson, Kristina M Sorensen, Brady J Ambrose, Jeffrey B Goodrich, Vern P Hart, Scott C Jensen, Hemang Patel, Leigh A Neumayer

**Affiliations:** 1 Department of Physics, Utah Valley University, Orem, UT 84058, USA; 2 Department of Pathology, University of Utah, Salt Lake City, UT 84112, USA; 3 Department of Surgery, University of Utah, Salt Lake City, UT 84132, USA; 4 Department of Mathematics and Statistics, Utah State University, Logan, UT 84322, USA; 5 Department of Physics, Utah State University, Logan, UT 84322, USA; 6 Department of Biomedical Engineering, Wayne State University, Detroit, MI 48202, USA

## Abstract

**Background:**

In addition to breast imaging, ultrasound offers the potential for characterizing and distinguishing between benign and malignant breast tissues due to their different microstructures and material properties. The aim of this study was to determine if high-frequency ultrasound (20-80 MHz) can provide pathology sensitive measurements for the *ex vivo *detection of cancer in margins during breast conservation surgery.

**Methods:**

Ultrasonic tests were performed on resected margins and other tissues obtained from 17 patients, resulting in 34 specimens that were classified into 15 pathology categories. Pulse-echo and through-transmission measurements were acquired from a total of 57 sites on the specimens using two single-element 50-MHz transducers. Ultrasonic attenuation and sound speed were obtained from time-domain waveforms. The waveforms were further processed with fast Fourier transforms to provide ultrasonic spectra and cepstra. The ultrasonic measurements and pathology types were analyzed for correlations. The specimens were additionally re-classified into five pathology types to determine specificity and sensitivity values.

**Results:**

The density of peaks in the ultrasonic spectra, a measure of spectral structure, showed significantly higher values for carcinomas and precancerous pathologies such as atypical ductal hyperplasia than for normal tissue. The slopes of the cepstra for non-malignant pathologies displayed significantly greater values that differentiated them from the normal and malignant tissues. The attenuation coefficients were sensitive to fat necrosis, fibroadenoma, and invasive lobular carcinoma. Specificities and sensitivities for differentiating pathologies from normal tissue were 100% and 86% for lobular carcinomas, 100% and 74% for ductal carcinomas, 80% and 82% for benign pathologies, and 80% and 100% for fat necrosis and adenomas. Specificities and sensitivities were also determined for differentiating each pathology type from the other four using a multivariate analysis. The results yielded specificities and sensitivities of 85% and 86% for lobular carcinomas, 85% and 74% for ductal carcinomas, 100% and 61% for benign pathologies, 84% and 100% for fat necrosis and adenomas, and 98% and 80% for normal tissue.

**Conclusions:**

Results from high-frequency ultrasonic measurements of human breast tissue specimens indicate that characteristics in the ultrasonic attenuation, spectra, and cepstra can be used to differentiate between normal, benign, and malignant breast pathologies.

## Background

In breast conservation surgery (BCS), obtaining negative (cancer free) margins is critically important for local control of breast cancer in the treated breast [1,2]. Consequently, failure to obtain negative margins during the initial surgery results in re-excision for 30-50% of patients [1-5]. A recent study of 994 women diagnosed with ductal carcinoma *in situ *(DCIS) showed that both treatment strategy (BCS alone, BCS with radiation therapy, or mastectomy) and margin status strongly correlated with long-term ipsilateral disease-free survival, but that positive or close margins following the last surgical treatment significantly reduced 5-year and 10-year ipsilateral event-free survival independent of treatment strategy [6].

Several approaches are therefore being investigated for the pre-operative and intraoperative estimation of margin sizes as well as for the intraoperative detection of cancer in surgical margins. Methods studied for the estimation of margin sizes include pre-operative CT and MRI and intraoperative ultrasonic imaging with conventional medical ultrasound instrumentation [4,7,8]. A number of electromagnetic and optical methods are also being developed for the intraoperative detection of cancer in margins. These include terahertz imaging [9], Raman spectroscopy [10], optical coherence tomography [11], and diffuse reflectance spectroscopy [12]. Intraoperative pathology methods currently being used for margin assessments include touch preparation cytology and frozen section analyses. These methods have limitations, however, including the requirement for an on-site trained pathologist, the inability to identify close margins (touch preparation cytology), and the ability to sample only a small portion of the margin (frozen section analyses) [12].

Many studies have shown that ultrasonic wave propagation in tissues is strongly dependent on histological features including cell structure, cell number density, tissue microstructure, and tissue heterogeneity [13-24]. Ultrasound therefore presents the potential of being able to differentiate between normal, benign, and malignant pathologies in breast tissue [25,26]. Of specific relevance to margin assessments was a study performed on eight mastectomy specimens using ultrasound transmission tomography from 2-10 MHz [27]. The frequency dependent attenuation was used to classify regions of each specimen into three types of tissue: Normal, benign changes, and invasive carcinoma. The high spatial resolution of the scans (≤ 1 mm) permitted a high degree of correlation to pathology micrographs, and yielded an 80% sensitivity, 90% specificity, and 86% accuracy for the three-way classification method.

High-frequency (HF) ultrasound has also been shown to be sensitive to changes in cell and tissue histology associated with mouse mammary tumors [22], apoptosis of malignant cells in centrifuged and dilute cell suspensions *in vitro *[28-30], apoptosis of malignant cells in rat tissues *ex vivo *and *in vivo *[31], and apoptosis in mouse tumors following photodynamic and radiation therapies [32,33]. Normal and malignant human breast epithelial cells have additionally been differentiated *in vitro *in monolayer cell cultures using 20-50 MHz ultrasound [34], and tumor size and margin status in 2-5 mm thick ductal carcinoma specimens have been determined with 15-50 MHz scanning acoustic microscopy [35].

In addition to experimental measurements, numerical models of ultrasonic wave propagation at the microstructural level have shown that HF ultrasound may be sensitive to tissue pathology [34,36-38]. Experimental studies using normal and malignant monolayer cultures of human breast epithelial cells as well as mouse liver specimens have validated the modeling approaches [34,38].

The objective of this study was to determine if HF ultrasound (20-80 MHz) could provide pathology sensitive measurements for the *ex vivo *detection of cancer in surgical margins obtained during breast conservation surgery. Both pulse-echo and through-transmission measurements were performed on the breast tissue specimens. The data analysis included examining conventional ultrasonic parameters such as ultrasonic sound speed and attenuation for correlations to pathology, as well as developing new approaches to analyze ultrasonic spectra and cepstra.

## Methods

**Figure 1:**
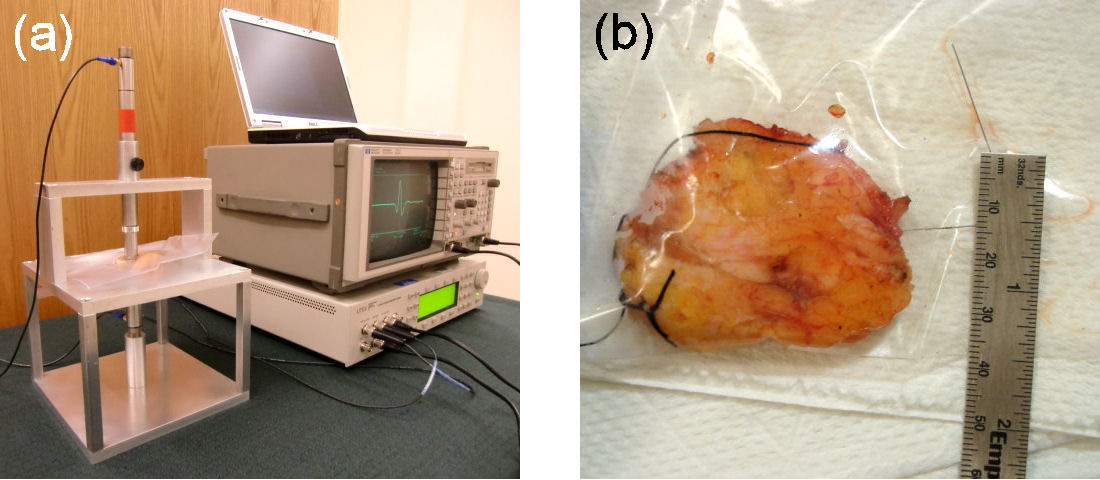
**Photograph of aluminum test fixture with instrumentation (a) and surgical margin in plastic re-sealable bag (b)**.

A HF ultrasonic test system, Figure 1a, was developed to collect simultaneous pulse-echo and through-transmission measurements from margins and other tissue specimens following resection from BCS. The data were analyzed with a variety of methods to search for correlations to tissue pathology.

### Clinical protocol

The ultrasonic testing of tissue specimens obtained during the course of routine breast conservation surgery was approved by the University of Utah Institutional Review Board on October 14, 2009, as a minimal risk study (IRB #00037350). Informed consent was obtained from patients for the use of their tissues for research purposes. Surgeries were performed on 17 patients by the collaborating surgeon and coauthor (LAN) at the Huntsman Cancer Hospital, Salt Lake City, Utah. The surgeries provided 34 resected specimens consisting of margins and other tissues such as lymph nodes and adenomas. The samples ranged from 1-5 cm in length and width, 0.1-1.5 cm in thickness, comprised a spectrum of both benign and malignant tissue pathologies, and did not require any additional procedures or resection that affected the patient or surgical outcome. Table 1 lists the range of pathologies provided by the specimens. For the purposes of this study the pathologies were categorized into 15 classifications. Immediately following resection, the surgeon placed each specimen inside a re-sealable plastic storage bag for ultrasonic testing (Figure 1b), and labeled the bag with a de-identified specimen number and, if applicable, the orientation of the margin.

**Table 1:** Pathology, number of specimens, and number of positions tested with high-frequency ultrasound

Tissue type	Specimens	No. of test positions
Lymph nodes (LN)	3	2
Benign or normal breast (BB)	4	5
Benign breast with calcifications (BC)	2	3
Atypical ductal hyperplasia (ADH)	2	5
Fibrocystic change (FC)	2	6
Fat necrosis (FN)	1	1
Fibroadenoma (FA)	2	2
Tubular adenoma (TA)	1	1
Papilloma (PA)	4	4

**Total benign**	**21**	**29**

Ductal carcinoma *in situ *(DCIS)	3	6
DCIS, solid and cribriform (DCIS-SC)	2	3
DCIS + IDC	3	9
Invasive ductal carcinoma (IDC)	2	3
Lobular carcinoma *in situ *(LCIS)	2	4
Invasive lobular carcinoma (ILC)	1	3

**Total Malignant**	**13**	**28**

Acronyms in parentheses are used in subsequent figures.

During the ultrasonic testing, the outside of the bag was coupled to the ultrasonic transducers with ultrasound scanning gel (Sonotech^® ^Clear Image). The surface moisture of the tissue provided sufficient coupling of the specimen to the inside of the bag for ultrasonic transmission. The bag therefore prevented contamination of the specimen with coupling fluid and additionally provided improved transmission of ultrasound between the transducers and specimen. One to four sites were tested on each specimen depending on the specimen size, resulting in a total of 57 sites tested. Triplicate waveforms were acquired from each test site on a specimen. After ultrasonic testing, routine pathology analyses were performed on the specimens. Ultrasonic results were correlated to pathology reports for each specimen.

### Ultrasonic materials and procedure

**Figure 2:**
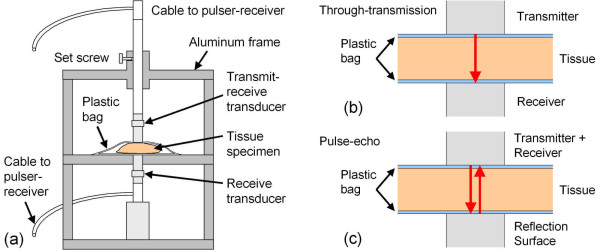
**Aluminum test fixture (a) and operation modes (b-c) used to position specimens and collect ultrasonic measurements**. In the pulse-echo mode (c), the receive transducer functions as a specular reflection surface for the ultrasonic waves.

Ultrasonic pulse-echo and through-transmission data were acquired from breast tissue specimens with the use of two immersion transducers (Olympus NDT, V358-SU, 50 MHz, 0.635-cm diameter active element), a HF square-wave pulser/receiver (UTEX, UT340), and a digital storage oscilloscope (Hewlett-Packard, HP-54522A, 500 MHz, 1Gs/s). Ultrasonic waveforms were averaged in the signal acquisition and downloaded onto a notebook PC using LabVIEW. The data acquisition parameters were pulse voltage = 100 V, pulse width = 10 ns, pulse repetition rate = 5 kHz, and receiver gain = 0-48 dB. An aluminum test fixture, Figure 2a, was used to support the tissue specimen, to position the transducers both above and below the sample for simultaneous pulse-echo and through-transmission measurements, and to lock the transducers into position. The thickness of the specimen was recorded for each ultrasonic measurement. A description was also recorded for each specimen, and photographs were taken of 19 specimens (e.g., Figure 1b).

The ultrasonic transducers each had a center frequency of 50 MHz and were broadband, providing a short pulse length and enhanced signal-to-noise in highly scattering or attenuating materials. The broadband characteristics of the transducers were also desired to obtain the ultrasonic response of the tissue across a wide frequency band.

### Ultrasonic data analysis

The HF ultrasonic signals acquired in this study were substantially different from the typical ultrasonic signals used for medical imaging, Doppler flow imaging, or tissue characterization. Whereas typical medical ultrasound signals are comprised of scattered waves from dispersed scattering centers, typically cells or nuclei, and other tissue inhomogeneities such as blood vessel walls, the signals collected in this study were of the transmitted pulse after propagating through the tissue specimen (through-transmission mode, Figure 2b) or of the specular reflection of the transmitted pulse from the surface of the second transducer (pulse-echo mode, Figure 2c). Therefore, in contrast to most medical ultrasound signals, the signals in this study had pulse-like characteristics with amplitudes significantly greater than background noise.

**Figure 3:**
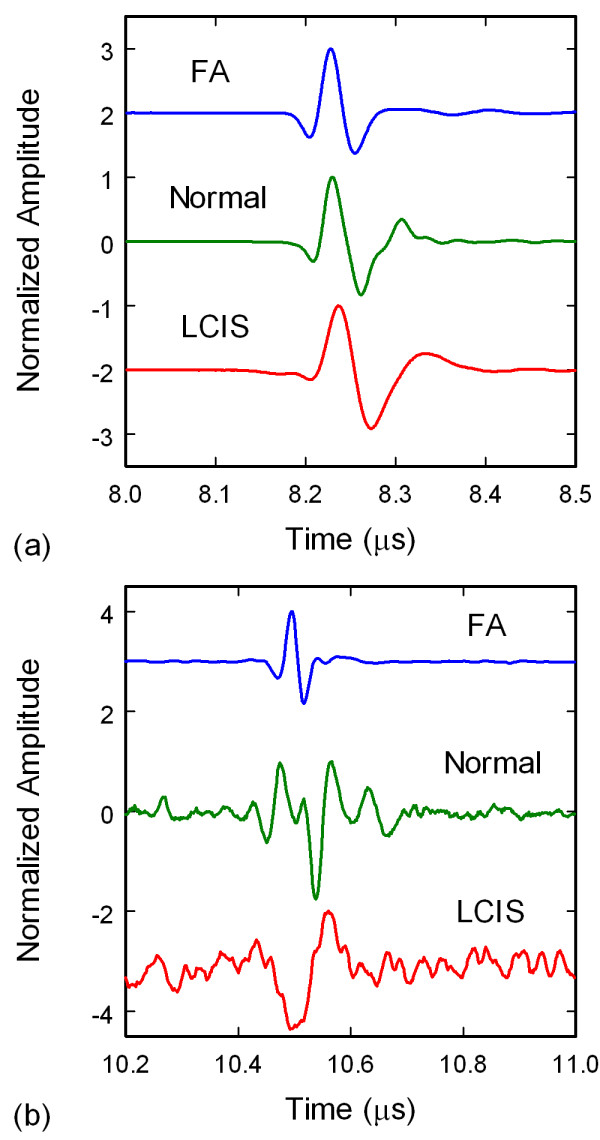
**Ultrasonic waveforms from through-transmission (a) and pulse-echo (b) measurements of surgical tissue specimens**. Amplitudes have been normalized and offset for comparison.

For through-transmission measurements, Figure 2b, the ultrasonic data consisted of time-domain waveforms of ultrasonic pulses, Figure 3a, that were transmitted from the top transducer, passed through the specimen only once, and received by the bottom transducer. For the pulse-echo measurements, Figure 2c, the ultrasonic data consisted of time-domain waveforms of ultrasonic pulses, Figure 3b, that were transmitted from the top transducer, passed through the specimen, reflected from the surface of the bottom transducer, passed through the specimen a second time, and received by the top transducer. The ultrasonic signals for both modes of operation therefore provided a convolution of the transducer and tissue responses.

Tumor progression and other atypical conditions affect the acoustic properties of tissues by altering the cell properties, the extracellular matrix properties, and the tissue microstructure. Measurement of sound speed and attenuation can therefore be used to reveal benign, pre-cancerous, or malignant tissues in breasts [25-27]. For calculation of ultrasonic sound speeds and attenuation coefficients, the arrival times and amplitudes of the time-domain waveforms were determined using a Hilbert transform. Arrival times were calibrated using a Plexiglas block as a substitute for the tissue samples. Attenuation coefficients were based on a relative scale by setting the lowest calculated attenuation value for the specimens (a fibroadenoma) to 0.003 Nepers/cm. Attenuation calculations accounted for receiver gain and specimen thickness.

**Figure 4:**
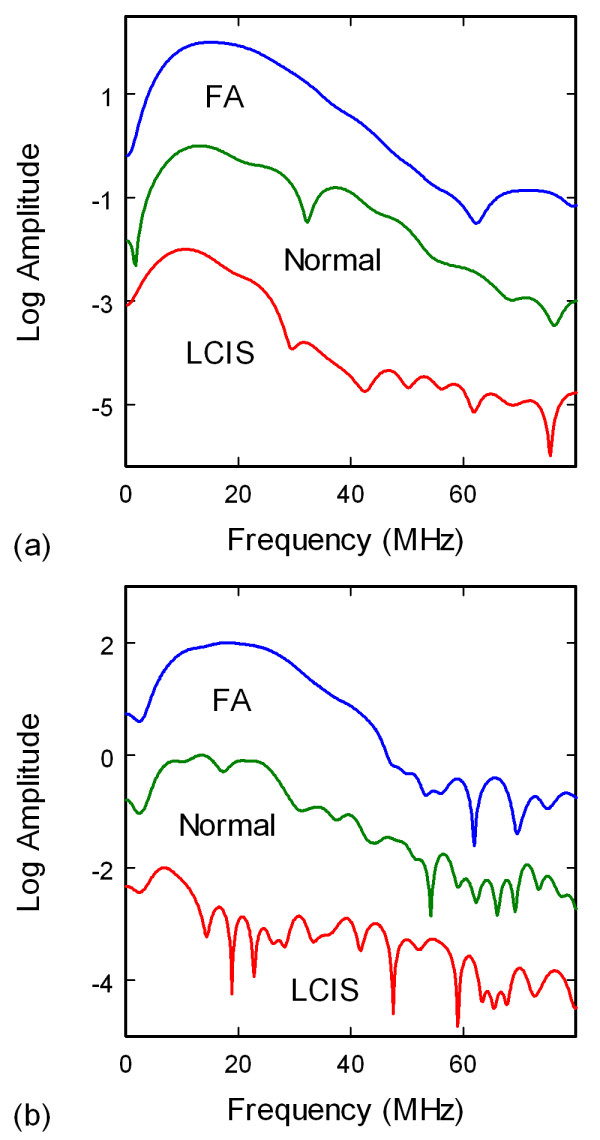
**Ultrasonic spectra from through-transmission (a) and pulse-echo (b) measurements of surgical tissue specimens**. Amplitudes have been normalized and offset for comparison. Note the increase in spectral structure (peaks and valleys) from FA to normal to LCIS, representing an increase in the ultrasonic parameter peak density.

The ultrasonic data were additionally analyzed in the frequency domain since previous numerical studies had indicated that the structure of HF ultrasonic spectra should be sensitive to neoplastic changes in breast tissues. Frequency spectra of the signals, Figures 4a and 4b, were obtained by subtracting background waveforms from the tissue waveforms, windowing the main signals in the waveforms, padding the waveforms to 4000 points to increase the spectral resolution, and performing a fast Fourier transform (FFT). The power spectra were then derived by taking the absolute value of the complex spectra. Analysis of the spectra included correlating specific spectral features, centroid frequencies of peak clusters, and the density of peaks and valleys. The density of peaks and valleys of a spectrum, from hereon referred to as the density of peaks or peak density, was calculated by counting the number of zero crossings of the derivative of the spectrum in the 20-80 MHz band.

The cepstrum is the inverse Fourier transform of the log power spectrum, and has been used to provide the mean scatterer spacing from ultrasonic data [20,39-41]. Applications have included measuring tibial cortical thickness and the location of brachytherapy seeds in tissue [40,41]. The cepstrum has also been used to obtain the mean scatterer spacing for breast tissue classified as benign, simple carcinoma, infiltrating papillary carcinoma, and fibroadenoma [20]. However, the low spectral range, 0-10 MHz, limited the measurement of scatterer spacings to greater than 0.15 mm, and the measured mean scatterer spacing varied from 0.82 ± 0.10 mm for normal breast tissue to 1.25 ± 21 mm for simple carcinoma.

The cepstra of waveforms were calculated in this study by computing the spectrum from the unpadded waveform, computing the inverse FFT of the log power spectrum, and then taking the absolute value of the resulting complex function. A modified cepstrum was also used in this study to analyze data. Computation of the modified cepstrum involved using the power spectrum derived from the padded waveform, and were obtained by windowing the power spectrum from 0 to 62.5 MHz, re-padding the spectrum to 4000 points, performing a second forward FFT on the padded spectrum, taking the absolute value of the complex function, and normalizing the curves. The results produced modified cepstra that showed a maximum at 0 μs and that sloped downward with multiple peaks at various positions. The modified cepstra were analyzed by calculating the slope of the log of the modified cepstrum, which was approximately linear in the 0-0.3 μs range. The value of the modified cepstrum at 0.3 μs was also calculated. The intercept at 0.3 μs was chosen as a measurement parameter due to the change in slope of the modified cepstrum at this point in the curve.

The data were evaluated with bar charts using the median for the bar height and the median absolute deviation (MAD) of the analyzed parameters for the error bars. After analyzing the data by the 15 pathology types as shown in Table 1, the data were reclassified into 5 pathology types: (1) normal breast tissue, (2) FN-FA-TA (fat necrosis, fibroadenoma, and tubular adenoma), (3) benign pathologies (BC, ADH, FC, and PA), (4) ductal carcinomas (DCIS, DCIS-SC, DCIS + IDC, and IDC), and (5) lobular carcinomas (LCIS and ILC). These categories were used to assess the efficacy of the preliminary measurements in this study for differentiating carcinoma in resected margins. Specificities and sensitivities for pathology types (2)-(5) were calculated with respect to normal tissue (1). Specificities and sensitivities for the five pathology types were additionally determined using a two-parameter multivariate analysis. Finally, *t*-tests and one-way ANOVA tests were performed to evaluate the significance level of the results.

## Results

### Sound speed and attenuation measurements

The ultrasonic sound speed measurements were widely scattered and displayed large deviations, rendering a differentiation of pathology types difficult. Since the time measurements were accurate to 1 ns (through-transmission) and 2 ns (pulse-echo), the principal cause for the sound speed variations was the error in the thickness measurements, which were performed manually by measuring the displacement of the search tube that held the top transducer from the test fixture. The error in this measurement was ± 0.5 mm, providing sound speed errors from 3.3% for the thickest samples (15.5 mm) to 42% for the thinnest samples (1.2 mm). Since the mean sample thickness was 5.0 mm, the average error in thickness and sound speed would be ± 10%. For glandular breast tissue, this error would translate to a sound speed measurement of approximately 1.52 ± 0.15 mm/μs [25,42]. Since the ultrasonic velocities of breast fat, cysts, and tumors lie within this range (1.46, 1.57, and 1.55 mm/μs, respectively) [25], it would be difficult to differentiate between different breast pathologies with sound speed measurements from this study.

**Figure 5:**
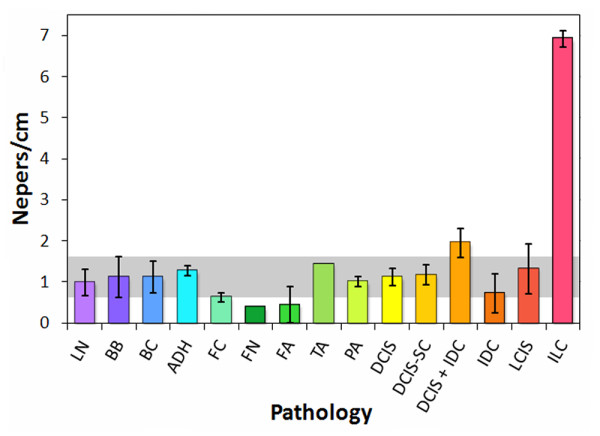
**Attenuation coefficients from through-transmission data of tissue specimens, classified by pathology type**.

The ultrasonic attenuation measurements were prone to similar large variations since the attenuation coefficient is inversely proportional to the thickness. Figure 5 displays the attenuation coefficients for the through-transmission data. As shown in Figure 5, the attenuation coefficients for most of the pathology classifications fall within the median absolute deviation range for the normal breast tissue (gray band). The exceptions are (1) fat necrosis and fibroadenoma, which fall below the gray band, (2) DCIS + IDC, which lies immediately above the gray band, and (3) ILC, with an attenuation substantially higher than all of the other pathologies and without overlapping deviations. These results are consistent with published data, which show lower attenuations for fat and cysts as compared to glandular breast tissue and considerably higher attenuations for tumors [25,42]. The attenuation coefficients for the pulse-echo data were less accurate due to the double pass of the wave through the sample and plastic bag, giving rise to additional reflection losses.

### Spectrum analysis

**Figure 6:**
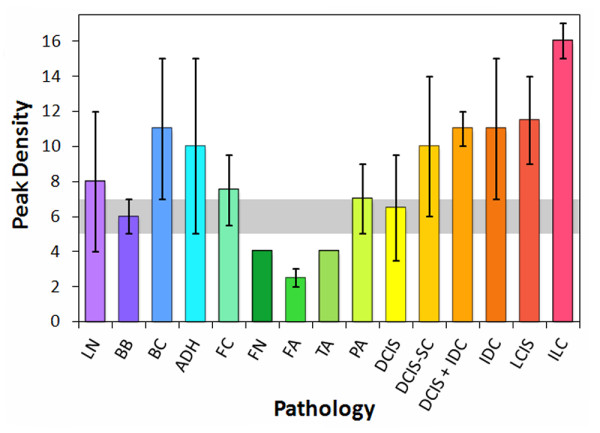
**Peak densities in the 20-80 MHz band of through-transmission spectra from tissue specimens**.

The ultrasonic spectra displayed a wide variation in structure that roughly corresponded to pathology. Although no single peak or group of peaks were found that could be used to differentiate tissue type, the total number of peaks and valleys in a specified spectral band appeared to be dependent on tissue pathology. Figure 6 shows the density of peaks and valleys for the 20-80 MHz spectral band for the through-transmission data. The peak density trends indicate that a majority of the carcinoma pathologies are above the median absolute deviation range for normal breast tissue (gray band), with ILC displaying the highest peak densities. The benign breast with calcifications and ADH classifications also show significant separation from the normal breast range, whereas the fat necrosis and adenoma specimens lie below the gray band. Pathologies involving intraductal or intralobular changes therefore show elevated peak densities, whereas those involving stromal proliferation (adenomas) or fat necrosis show decreased peak densities. The peak densities in the 0-50 MHz band showed similar trends as the 20-80 MHz band, but with greater deviations. The peak densities from the pulse-echo data displayed less consistent trends that were less useful at distinguishing between different pathology types.

### Cepstrum analysis

A cepstrum analysis of the pulse-echo data showed that several of the samples produced multiple peaks across a range of mean scatterer spacings *d *= *ct*/2, where *d *is the spacing between scatterers, *c *is the tissue sound speed, and *t *is the time of the peak in the cepstrum [39]. Most of the peaks occurred in an apparently random fashion and could not be correlated to pathology. However, one peak at *t *= 0.102 μs (*d *= 77 μm) occurred prominently in 10 of the 15 pathology types, but was absent in lymph node, fibroadenoma, tubular adenoma, DCIS + IDC, and LCIS tissues. In the 10 pathology types where the peak was present, the amplitude of the peak varied significantly from specimen to specimen, and it therefore could not be used to discriminate between the 10 pathology classifications. A secondary peak at *t *= 0.2 μs was additionally present whenever the 0.102-μs peak was observed, indicating that the 0.2-μs peak was due to either a multiple wave reflection or a multiple of the mean scatterer spacing.

**Figure 7:**
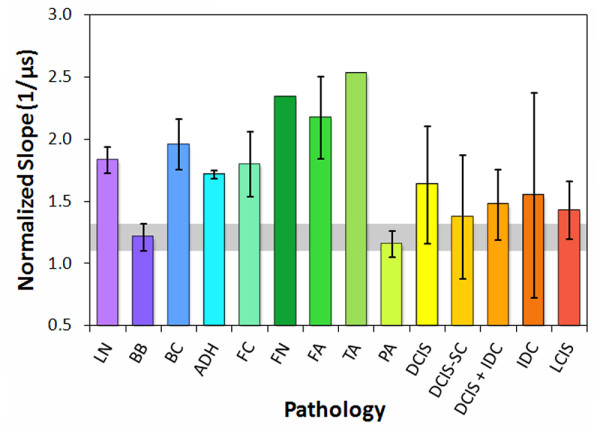
**Modified cepstrum slopes from pulse-echo data of tissue specimens, classified by pathology type**.

Since the slopes of the modified cepstra from 0 to 0.3 μs were negative, the absolute values of the slopes are displayed in Figure 7 for comparison of trends. Intraductal papilloma displayed essentially the same slope and deviation values as normal breast tissue. The carcinomas displayed slopes above the median absolute deviation range for normal breast tissue (gray band), but their large deviations indicated poor separation from the normal breast tissue values. However, the other seven benign pathologies and tissues displayed significantly greater slopes than normal breast tissue, with values and deviations well above the normal breast tissue range. Fat necrosis, fibroadenoma, and tubular adenoma displayed the greatest slopes. The modified cepstrum values at 0.3 μs produced trends similar to the slopes.

### Results for re-categorized pathology types

**Figure 8:**
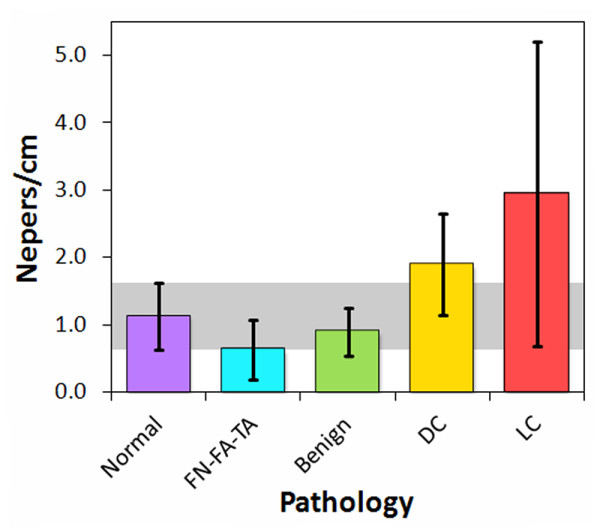
**Attenuation coefficients for the reclassified tissue specimens**.

**Figure 9:**
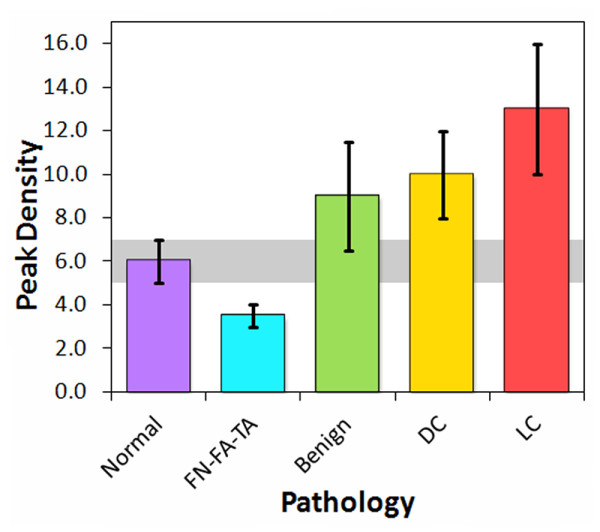
**Peak densities in the 20-80 MHz band for the reclassified tissue specimens**.

**Figure 10:**
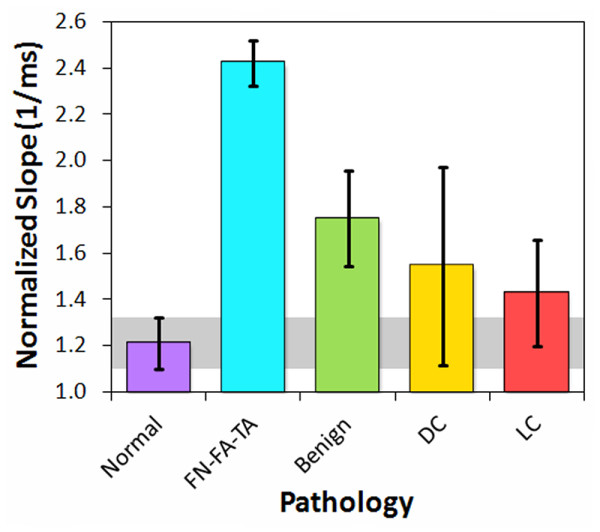
**Modified cepstrum slopes for the reclassified tissue specimens**.

By reclassifying the breast pathologies into five groups, the efficacy of the analysis parameters and high-frequency ultrasonic data used in this study were assessed for the detection of carcinoma in resected margins. The reclassified pathology types were (1) normal breast tissue, (2) fat necrosis/fibroadenoma/tubular adenoma (FN-FA-TA), (3) benign pathologies, (4) ductal carcinomas (DCIS and IDC), and (5) lobular carcinomas (LCIS and ILC). Figures 8, 9, and 10 show the attenuation coefficients, peak densities, and cepstral slopes, respectively, for the reclassified pathology types.

Table 2 displays a preliminary analysis of the data shown in Figures 8, 9, and 10 using binary classification tests to yield the specificity and sensitivity of each tissue category as compared to normal breast tissue. The specificity and sensitivity values were calculated directly from the measured data. The mean of the median values was used as the classification threshold between each tissue category and normal tissue. The peak density provided the highest values between normal and malignant tissues, whereas the cepstrum slope provided the highest values between normal tissue and benign pathologies. Both peak density and cepstrum slope gave the same values between normal and FN-FA-TA pathologies. The binary classification tests indicated higher specificities and sensitivities for lobular carcinomas than for ductal carcinomas. The specificities and sensitivities are expected to improve with more measurements from future studies.

**Table 2:** Highest specificity and sensitivity values from analysis of data classified into five pathology categories

Pathology	Parameter	Specificity	Sensitivity
Lobular carcinomas	Peak density	100%	86%
Ductal carcinomas	Peak density	100%	74%
FN-FA-TA	Peak density & cepstrum slope	80%	100%
Benign pathologies	Cepstrum slope	80%	82%

The specificity and sensitivity for each tissue category was calculated with respect to normal breast tissue.

The significance of the specificities and sensitivities in Table 2 were analyzed with *t*-tests. Table 3 displays the *t*-test and *p*-value for each of the four pathology groups in comparison to normal tissue, and for each of the three ultrasonic parameters. The analyses that provided statistically significant values (*p *< 0.05) were peak density for lobular carcinomas, ductal carcinomas, and FN-FA-TA pathologies, and cepstrum slope for FN-FA-TA and benign pathologies. All five pathology groups were additionally analyzed with one-way ANOVA tests to determine which of the three ultrasonic parameters provided statistically significant separation of all five groups. The *F*-ratio for attenuation was *F*_4, 31 _= 3.933, indicating that the results are significant at the 5% level and very close to the 1% level of significance. Similarly, the *F*-ratio for peak density was *F*_4, 31 _= 3.728, again indicating that the results are significant at the 5% level and close to the 1% level of significance. Finally, the *F*-ratio for cepstrum slope was *F*_4, 25 _= 1.854, indicating that the results are not significant at the 10% level. Therefore, in contrast to the paired *t*-tests, the ANOVA tests suggest that attenuation and peak density provide the highest significance for distinguishing between the pathology types.

**Table 3:** *t*-test results from analysis of data classified into five pathology categories

Pathology	Attenuation	Peak density	Cepstrum slope
Lobular carcinomas	*t*(10) = 2.14 *p *< 0.10	*t*(10) = 2.952 *p *< 0.02	*t*(10) = 0.88 *p *> 0.20
Ductal carcinomas	*t*(22) = 1.305 *p *> 0.20	*t*(22) = 2.233 *p *< 0.05	*t*(19) = 1.406 *p *< 0.20
FN-FA-TA	*t*(7) = 1.278 *p *> 0.20	*t*(7) = 2.609 *p *< 0.05	*t*(7) = 4.615 *p *< 0.01
Benign pathologies	*t*(21) = 1.414 *p *< 0.20	*t*(21) = 1.751 *p *< 0.10	*t*(20) = 2.883 *p *< 0.01

The *t*-test and *p*-value for each tissue category was calculated with respect to normal breast tissue.

**Figure 11:**
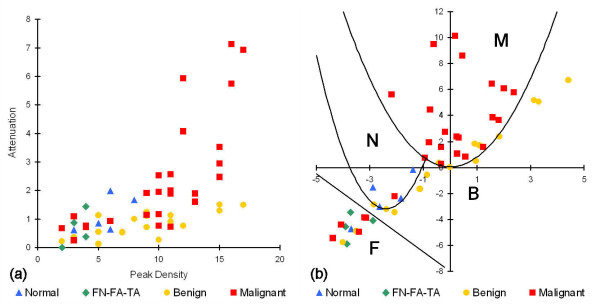
**Multivariate analysis of peak density and attenuation parameters in ultrasonic data**. (a) Non-rotated plot of attenuation vs. peak density. (b) Rotated and translated plot of attenuation vs. peak density, showing the use of parabolic and linear curves for the classification boundaries. F = fat necrosis - fibroadenoma - tubular adenoma. B = benign pathology. N = normal breast tissue. M = malignant breast tissue.

A multivariate analysis was also performed on the re-categorized data by using the two-dimensional parameter space defined by attenuation and peak density (Figure 11a). Classification boundaries were determined in this space by rotating and translating the coordinates of the data points and calculating linear and parabolic boundaries that maximized inclusion of a pathology category and exclusion of the other four categories (Figure 11b). The one exception was for ductal and lobular carcinomas, which were intimately mixed and therefore difficult to separate in this first-look analysis. Specificities and sensitivities for each of the five pathology types, Table 4, were then calculated with respect to all of the other pathology types that were excluded by the boundary. The multivariate analysis shows that the ultrasonic measurements have good specificity and sensitivity for carcinomas with respect to all benign conditions (normal breast tissue, benign pathologies, and FN-FA-TA).

**Table 4:** Multivariate analysis results

Pathology	Specificity	Sensitivity
Lobular carcinomas	85%	86%
Ductal carcinomas	85%	74%
FN-FA-TA	84%	100%
Benign pathologies	100%	61%
Normal tissue	98%	80%

The specificity and sensitivity for each tissue category was calculated with respect to a five-way classification system using the classification boundaries as defined in Figure 11.

The sensitivities for the carcinomas and FN-FA-TA pathologies remained the same in the multivariate analysis, whereas the specificities for the FN-FA-TA and benign pathologies increased. Values that decreased in the multivariate analysis included the specificities for the carcinomas and the sensitivity for the benign pathologies. Although some of the values in Table 4 are lower than those in Table 2, this is to be expected since Table 2 reports values for detecting and differentiating a particular pathology from only normal tissue, whereas Table 4 reports values for detecting and differentiating a particular pathology from all other studied pathology types. The overlap between pathology categories is therefore more evident in the multivariate analysis, and consequently the results in Table 4 are more realistic for distinguishing between pathologies such as ductal carcinoma and benign pathologies (e.g., ADH or fibrocystic changes).

The specificity and sensitivity results from this study (Tables 2 and 4) are comparable to those for various methods currently in use or under development for intraoperative margin assessments. Table 5 summarizes the reported specificity and sensitivity values for several of these methods. Since the values in Table 5 are primarily for malignant versus normal breast tissue, they are comparable most properly to the values in Table 2.

**Table 5:** Specificity and sensitivity values for various intraoperative margin assessment methods

Method and references	Specificity	Sensitivity
Touch preparation cytology [45,46]	83-100%	75-96%
Frozen section analysis [5,47,48]	92-100%	65-78%
Near-field RF spectroscopy [49]	70%	70%
Raman spectroscopy [50]	93%	83%
Optical coherence tomography [11]	82%	100%
Fluorescence and reflectance spectroscopy [51]	96%	85%
Low-freq. (2-10 MHz) ultrasonic attenuation [27]	90%	80%

Values represent comparison between normal vs. malignant tissue.

A principal advantage of the HF ultrasonic method reported in this study over several of the methods listed in Table 5 is its ability to differentiate across a wider class of breast pathologies, including benign conditions and fat necrosis-adenomas. The ability to differentiate between different types of breast pathology, including different types of breast cancer, would be a significant advantage for an intraoperative margin assessment method. Of particular importance would be the capability to distinguish benign pathologies such as ADH and fibrocystic changes from malignancies. Although a basic multivariate analysis of our preliminary data does not yet provide high enough sensitivities and specificities (> 70%) for clinically relevant detection and differentiation of all five pathology categories (specifically for benign pathologies), refinement of the measurement technique and multivariate analyses of larger, more comprehensive data sets may improve these capabilities. They may also provide further diagnostic capabilities for a more highly resolved classification system such as shown in Table 1 and Figures 5, 6, and 7.

The strong response of HF ultrasound to lobular carcinomas (Table 2 and Figures 5, 6, 8, and 9) may additionally provide an accurate and clinically important method to detect ILC in surgical margins. Negative margins are difficult to achieve for ILC with conventional BCS. Six studies published between 1994 and 2006 reported 49-63% positive or close margins following the initial surgery, and a recent study reported the use of full thickness excision and oncoplastic surgery to lower the rate of positive/close margins to 39% [43]. Taken as a pathology classification by itself, the findings of our study show that ILC is particularly easy to detect and identify as compared to other carcinomas and pathologies. Both peak density and attenuation provide specificity and sensitivity values of 100% for differentiating ILC from normal breast tissue. Attenuation also has 100% specificity and sensitivity for differentiating ILC from benign pathologies, whereas peak density has 83% specificity and 67% sensitivity.

## Discussion

### Correlation of results and microstructural interpretations

**Figure 12:**
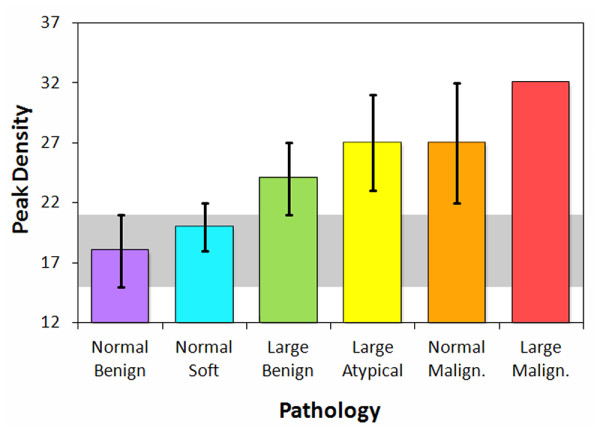
**Simulated peak densities in the 20-80 MHz spectral band for a layered cylinder model**. Model consisted of arbitrarily oriented layered cylinders with a fluid-filled (benign or soft) or solid-filled (atypical or malignant) lumen.

Contrary to previous results from numerical models [36,37], no single peak or feature could be identified in the experimental spectra that correlated to pathology type and could therefore be used as a predictor for tissue microstructure. One parameter, however, that correlated with both benign and malignant changes to the mammary ducts was the spectral density of peaks. Pathologies that would result in enlargement of the duct or growth of a solid mass within the duct produced greater peak densities than normal breast tissue. Such pathologies included calcifications, ADH, intraductal papilloma, and DCIS solid and cribriform. These results appear to correlate strongly with the peak densities from ultrasonic backscatter spectra from a layered cylinder model, where duct enlargement or neoplasm growth in the lumen results in higher peak densities (Figure 12). The layered cylinder model used multipole expansions to simulate ultrasonic scattering from mammary ducts represented as three-dimensional cylinders with an epithelial cell layer and interior lumen, and was similar to a model used to simulate elastic wave scattering from normal and clotted blood vessels [44]. The observed increases in peak density with the layered cylinder model provide an interpretation of the experimental data in terms of microstructural remodeling of the normal ductal architecture. Increases in ductal diameter, wall thickness, and lumen composition (fluid, hyperplastic, or malignant) have a direct and significant affect on the peak densities.

A microstructural interpretation for the slope of the modified cepstrum is that the slope would be a measure of the distribution of scatterer spacings between 0 and 225 μm, with a large slope indicating a distribution skewed to small spacings, and a small slope indicating a distribution skewed to large spacings. The interpretation for the 0.3-μs intercept would be similar. Since the cepstra were normalized and had negative slopes, a high intercept value would indicate a shallow (small) slope and large scatterer spacings. Conversely, a low intercept value would indicate a steep (large) slope and small scatterer spacings. Figure 7 reveals that the slopes for the modified cepstra displayed significant differences for seven of the benign pathology types as compared to the normal breast tissue and carcinoma pathologies.

At first the cepstral results appear inconsistent with a histological interpretation. Ductal dilation, thickening, and hyperplasia are characteristic of several benign pathologies including calcifications, ADH, and fibrocystic changes. These changes are expected to increase the mean spacing of the scatterers, yet the cepstral results for the ultrasonic measurements indicate that the mean scatterer spacings are less for the benign pathologies. An alternative explanation, however, is that the expansion arising from ductal dilation will decrease the interductal spacings in the tissue. This interpretation attributes the mean scatter spacing, as measured by the modified cepstrum slope and 0.3-μs intercept, to the distances between neighboring ducts. This interpretation appears consistent with the experimental data. Further simulation work with models containing multiple layered cylinders with a range of microstructures and material properties may provide a more complete correlation of the cepstrum results to ductal architecture.

### Differentiation of pathology categories

The results of this pilot study indicate that high-frequency ultrasound can produce clinically relevant specificity and sensitivity values for detecting malignant tissues in surgical margins and differentiating them from normal tissue (Table 2) as well as from fat necroses, fibroadenomas, and tubular adenomas (Table 4). The sensitivity values for benign pathologies such as ADH, benign calcifications, fibrocystic change, and papilloma are low (< 70%), however, and are therefore not yet sufficient for differentiating these tissues from malignant tissues. These values may improve with a more rigorous multivariate analysis of the parameters obtained in this study from the ultrasonic waveform (attenuation), spectrum (peak density), and modified cepstrum (cepstral slope).

A single ultrasonic parameter is often insufficient to diagnose breast cancer *in vivo*, and many researchers are exploring multivariate methods to discriminate between malignant and benign pathologies in methods such as ultrasonic tomography [25,26]. Sound speed and attenuation have been the two most widely used parameters to date to combine into a multivariate analysis. The results of this study, however, indicate that attenuation, spectral peak density, and modified cepstrum slope may be complementary parameters for differentiating various breast pathologies.

The peak density results (Figures 6 and 12) indicate that disrupted ductal architectures produce higher peak densities in selected frequency ranges as compared to normal breast tissue. Exceptions to this correlation are the fat necrosis and adenomas which show lower peak densities than normal breast tissue and where ductal structures are either absent or severely distorted, respectively. Since both benign and malignant processes can disrupt ductal microstructures, a second parameter is required to differentiate between these two processes. The slopes or 0.3-μs intercepts of the modified cepstra (Figure 7) may provide this parameter by separating most of the benign pathologies from normal breast tissue and various carcinomas.

### Origin of uncertainties

As already discussed, one source of uncertainty in the experimental data was the measurement of tissue thickness, which has the most significant impact on the measurement of effective material properties such as sound speed and attenuation. The other source of uncertainty in the measurements was the correlation of the measurement position on the specimen to the microscopic extent of the pathology in the tissue. Although ultrasonic measurements were correlated to the orientation of the margin, the diameter of the transducer elements (0.635 cm), in addition to the lack of an exact point-by-point matching of transducer position to specimen pathology in this study, most likely resulted in the sampling of tissues of mixed pathologies (e.g., normal breast plus DCIS) in a significant number of measurements. This measurement uncertainty is most probably the main source of the median absolute deviations in the peak density and cepstrum plots (Figures 6 and 7). Finally, the small number of tested samples in this pilot study limits the statistical robustness of the results, particularly for pathology types with only one or two measurements. Implementing a more comprehensive experimental design in subsequent studies is therefore essential to minimizing the thickness and positioning errors as well as to increasing the number of measurements for each pathology category.

## Conclusions

High-frequency ultrasonic measurements were collected from resected margins and other breast tissues. Attenuation, spectral, and cepstral analyses of these measurements show correlations to both benign and malignant pathologies that could potentially be used in a multivariate analysis to determine tissue pathology for intraoperative margin assessments. The density of peaks in the ultrasonic spectra is a key parameter in the correlations, and appears to be linked to the disruption of the ductal architecture in breast tissue.

## List of abbreviations

ADH: atypical ductal hyperplasia; BB: benign breast; BC: benign breast with calcifications; DC: ductal carcinoma; DCIS: ductal carcinoma *in situ*; DCIS-SC: ductal carcinoma *in situ*, solid and cribriform; FA: fibroadenoma; FC: fibrocystic change; FN: fat necrosis; HF: high-frequency; IDC: invasive ductal carcinoma; ILC: invasive lobular carcinoma; LC: lobular carcinoma; LCIS: lobular carcinoma *in situ*; LN: lymph node; PA: papilloma; TA: tubular adenoma

## Competing interests

TED and LAN have applied for a patent relating to the content of this manuscript, but have not received reimbursements, fees, funding, or salary from an organization that holds or has applied for patents relating to the content of this manuscript. REF, CLE, KMS, BJA, JBG, VPH, SCJ, and HP declare that they have no competing interests.

## Authors' contributions

TED participated in the study design, methods development, data acquisition, data analysis, interpretation of results, and drafting of the manuscript. REF participated in the data acquisition, interpretation of results, and data analysis. CLE participated in the data acquisition. KMS participated in the data analysis, interpretation of results, and preparation of the manuscript. BJA designed the test fixture for the data acquisition and wrote the data acquisition software. JBG, VPH, SCJ, and HP participated in the data analysis. LAN participated in the study design, data acquisition, and preparation of the manuscript. All authors read and approved the final manuscript.

## Authors' information

TED is Assistant Professor of Physics at Utah Valley University and Research Associate Professor of Physics at Utah State University. His research includes developing computational and experimental methods in ultrasonics, biomechanics, and optics for the study, detection, and treatment of cancer.

REF is Assistant Professor of Pathology at the University of Utah and Huntsman Cancer Institute. She practices general surgical pathology and cytology with an interest in breast pathology and cytopathology.

CLE is a medical student at the University of Utah School of Medicine. She has worked in genetic research involving hereditary breast cancer, and her interests include surgery and genetics.

KMS is a mathematics student at Utah State University performing research in acoustics, signal analysis, and medical ultrasound.

BJA is a graduate of Utah State University in Physics and is currently working as a research and development engineer. His interests are in streamlining development processes for new products.

JBG is a graduate of Utah State University in Physics. His interests are in medical physics.

VPH is a Ph.D. candidate in Physics at Utah State University performing research in the development of tomographic and computational methods for upper atmospheric studies and medical physics.

SCJ is a Physics student at Utah State University performing research in biophysics, nanophysics, and nanomedicine.

HP is a postdoctoral fellow in Biomedical Engineering at Wayne State University. His research interests are in tissue engineering.

LAN is Professor of Surgery at the University of Utah School of Medicine, and is a member of the multidisciplinary team treating breast cancer at the Huntsman Cancer Institute. She holds a Jon and Karen Huntsman Presidential Professorship in Cancer Research, has more than 15 years of experience with skin-sparing mastectomy, and has six years of experience with sentinel lymph node biopsy.

## Pre-publication history

The pre-publication history for this paper can be accessed here:

http://www.biomedcentral.com/1471-2407/11/444/prepub
